# Widespread folate receptor expression in pediatric and adolescent solid tumors - opportunity for intraoperative visualization with the novel fluorescent agent pafolacianine

**DOI:** 10.18632/oncotarget.28772

**Published:** 2025-10-16

**Authors:** Ashley C. Dodd, Nitin R. Wadhwani, Alison Lehane, Rom Brown, Kyle L. MacQuarrie, Seth D. Goldstein, Timothy B. Lautz

**Affiliations:** ^1^Department of Surgery, Ann & Robert H. Lurie Children’s Hospital, Chicago, IL 60611, USA; ^2^Department of Pathology and Laboratory Medicine, Ann & Robert H. Lurie Children’s Hospital, Chicago, IL 60611, USA; ^3^Stanley Manne Children’s Research Institute, Ann & Robert H. Lurie Children’s Hospital of Chicago, Chicago, IL 60611, USA; ^4^Division of Pediatric Hematology, Oncology, and Stem Cell Transplantation, Ann & Robert H. Lurie Children’s Hospital, Chicago, IL 60611, USA; ^5^Division of Pediatric Surgery, Department of Surgery, Northwestern University Feinberg School of Medicine, Chicago, IL 60611, USA

**Keywords:** folate receptor, fluorescent-guided surgery, pediatric tumors, pafolacianine, RNA sequencing

## Abstract

Contemporary fluorescence-guided surgery has evolved principally based on the uses and limitations of the contrast agent indocyanine green (ICG). A second generation of novel fluorescent agents are under development to target specific molecular markers on tumor cells and/or the tumor micro-environment. Pafolacianine, a molecular agent targeting the folate receptor (FR), is the first of these approved for use in adults, but its potential utility in pediatric cancers is unknown. In this study, we performed immunohistochemistry staining on slides obtained from a range of pediatric patients with solid tumors. Slides were stained with antibodies to FRα and FRβ, and fluorescence was quantified. Separately, publicly available RNA sequencing data were queried for both FRα and FRβ expression in various pediatric tumors.

## INTRODUCTION

The field of fluorescence-guided surgical oncology has expanded rapidly in the past decade, driven by efforts to facilitate negative-margin resection, identification of regional lymph nodes, and protection of adjacent normal structures among other indications [1–3]. The vast majority of current applications of fluorescence-guided surgery utilize indocyanine green (ICG), which has well-established uses for fluorescence cholangiography, sentinel lymph node evaluation, assessment of tissue perfusion, and a growing body of experience for tumor identification and margin delineation [4–6]. However, most oncologic applications of ICG rely on the relatively non-specific phenomenon of “enhanced permeability and retention”, wherein tumor tissues have increased uptake and decreased lymphatic drainage of albumin-bound ICG causing relatively increased uptake and retention. This is relatively non-specific and largely dependent on vascular permeability and tissue characteristics [7–9].

Accordingly, there is great translational interest in the development of target-specific fluorophores for tumor localization at both primary and metastatic sites. These fluorophores will ideally bind to tumor-specific surface proteins or target unique features of the tumor microenvironment and thus allow for precise distinction between tumor and surrounding normal tissue. Pafolacianine (Cytalux^™^; On Target Laboratories, Inc.) is an intravenously administered drug that targets folate receptors (FRα and FRβ) [7, 9, 10]. The fluorophore is a folate analog conjugated to a near infrared (NIR) dye. Binding of the folate receptor triggers endocytosis of the receptor/fluorophore, which allows it to concentrate intracellularly. When illuminated with a NIR camera, the fluorophore becomes excited and emits a fluorescence that is detected using the camera system [11, 12].

Folate receptors are over-expressed on many solid tumors, including most ovarian carcinomas, for which pafolacianine was first FDA approved. It has been additionally approved for adult tumors in the lung and remains an active investigational agent in other folate-expressing tumors [13–15]. In this study, we explored the expression of folate receptors in various pediatric solid tumors in order to identify potential pafolacianine targets in children.

## RESULTS

We reviewed FRα and FRβ immunostains that were performed on formalin fixed paraffin embedded tissue samples consisting of 19 specimens from 13 pediatric and adolescent patients with solid tumors (Table 1).

**Table 1:** Tumor nodule characteristics and folate receptor expression

Subject identifier	Age (years)	Histology	Location	Primary or metastatic	FRα Score	FRβ Score	FRβ expression location (T, ME, B)
1	7	Wilms Tumor	Kidney	Primary	0	3	B
	8	Wilms Tumor	Lung	Metastatic	0	2	B
2	16	Wilms Tumor	Kidney	Primary	0	1	TME
	17	Wilms Tumor	Chest wall	Metastatic	0	2	B
3	14	Osteosarcoma	Femur	Primary	0	2	TME
	15	Osteosarcoma	Lung	Metastatic	1	3	T
4	17	Synovial sarcoma	Calf	Primary	1	3	B
	19	Synovial sarcoma	Lung	Metastatic	0	3	B
5	2	Rhabdomyosarcoma	Abdomen/bladder	Primary	0	3	T
		Rhabdomyosarcoma	Abdomen	Primary	0	1	TME
6	12	Desmoplastic small round cell tumor	Abdomen	Primary	0	2	B
		Desmoplastic small round cell tumor	Chest	Primary	0	3	B
7	2	Rhabdomyosarcoma	Abdomen	Primary	0	1	B
8	18	Ewing sarcoma	Lung	Metastatic	0	3	B
9	21	Osteosarcoma	Lung	Metastatic	0	3	T
10	16	Desmoplastic small round cell tumor	Abdomen	Primary	0	3	B
11	18	Osteosarcoma	Lung	Metastatic	0	3	B
12	20	Ewing sarcoma	Lung	Metastatic	1	2	B
13	5	Neuroblastoma	Lung	Metastatic	0	3	T

For FRβ expression location: T: Tumor, TME: Tumor Microenvironment, B: Both tumor and the tumor microenvironment.

### FRα

FRα demonstrated only nominal expression and had a predominantly weak expression across our specimen samples (Table 2). Only 16% of the analyzed specimens demonstrated FRα expression, but none of the specimens demonstrated strong levels of staining. Only osteosarcoma, synovial sarcoma, and Ewing sarcoma showed weak FRα expression.

**Table 2:** Comparison of immunohistochemistry (IHC) staining intensity of FRα and FRβ

Receptor	No staining	Weak staining	Moderate staining	Strong staining
**FRα**	16	3	0	0
**FRβ**	0	3	5	11

### FRβ

FRβ demonstrated universal tumor expression. Not only was it present in 100% of analyzed specimens, but 58% of specimens had the highest intensity (3) of expression. Only 3 of the specimens, a primary Wilms tumor and two primary Rhabdomyosarcomas, showed weak FRβ expression. Interestingly, a metastatic lesion from the same patient with a weakly-expressing Wilms tumor demonstrated moderate FRβ expression within the metastatic lesion. The remaining Wilms tumors showed moderate to strong FRβ expression, along with strong FRβ expression in osteosarcoma (75%), synovial sarcoma (100%), rhabdomyosarcoma (33%), DSRCT (33%), Ewing sarcoma (50%), and neuroblastoma (100%).

**Figure 1:**
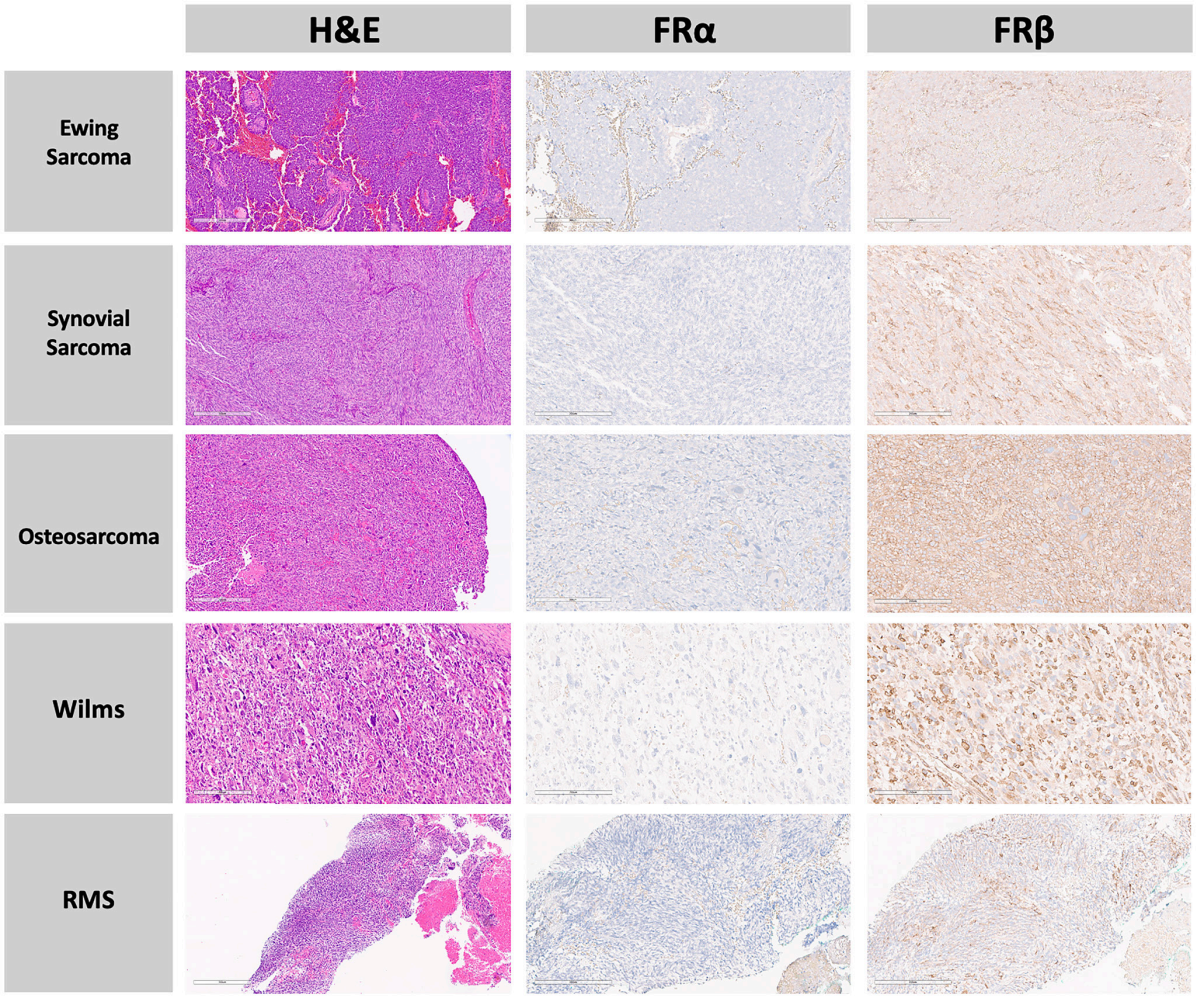
Panel of various pediatric tumors comparing H&E staining, FRα, and FRβ protein expression within the tumor. None of these tumors demonstrate FRα expression; however all of the tumors demonstrate moderate to strong FRβ expression (brown staining). Row 1: Ewing Sarcoma in a pulmonary metastatic lesion of a 20-year-old. Row 2: Primary Synovial Sarcoma in a 17-year-old. Row 3: Osteosarcoma in a pulmonary metastatic lesion of a 15-year-old. Row 4: Primary Wilms tumor in a 7-year-old. Row 5: Primary Rhabdomyosarcoma (RMS) in a 2-year-old. H&E slides at 10x magnification. FRα and FRβ stained slides at 20x magnification.

**Figure 2:**
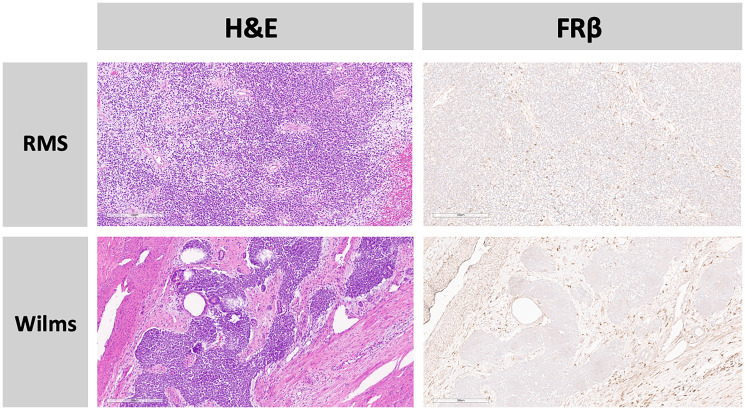
FRβ protein expression in the tumor micro-environment alone. Row 1: Primary Rhabdomyosarcoma (RMS) in a 2-year-old. Row 2: Primary Wilms tumor in a 16-year-old. H&E slides and FRβ stained slides at 10x magnification.

**Figure 3:**
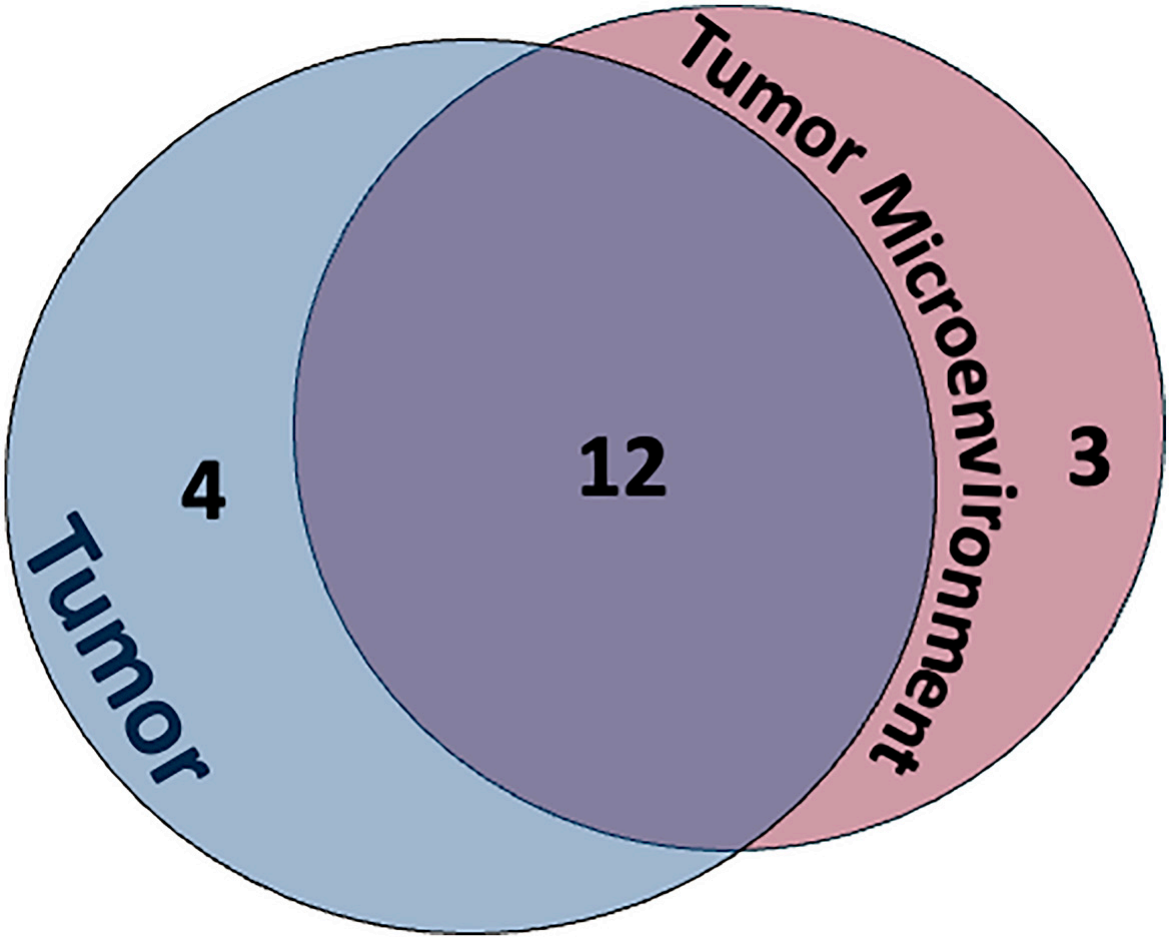
Venn Diagram demonstrating overlap of FRβ protein expression in the tumor itself and within the tumor microenvironment (TME). 63% had FRβ protein expression in both the tumor and the surrounding TME.

The expression of FRβ was present both on the tumor cell surface (Figure 1) and within the tumor microenvironment (Figure 2). FRβ was expressed on the cell surface in 84% of samples and within the tumor micro-environment in 79% of samples, with some samples having both (Figure 3). Among 11 slides with normal tissue present (8 lung, 1 kidney, 1 muscle, and 1 bladder), there was a predominantly weak FRβ expression within the normal tissue, most commonly in the lungs/pleura (73%). 64% of the slides with normal tissue present showed strong expression on the tumor cell surface, within the micro-environment, or both, allotting for a distinction between the tumor and surrounding normal tissue. In all but one of those slides we were able to distinguish a difference between normal tissue and the tumor/tumor micro-environment based on FRβ expression. The one slide we were not able to easily distinguish lacked sufficient normal tissue.

**Figure 4:**
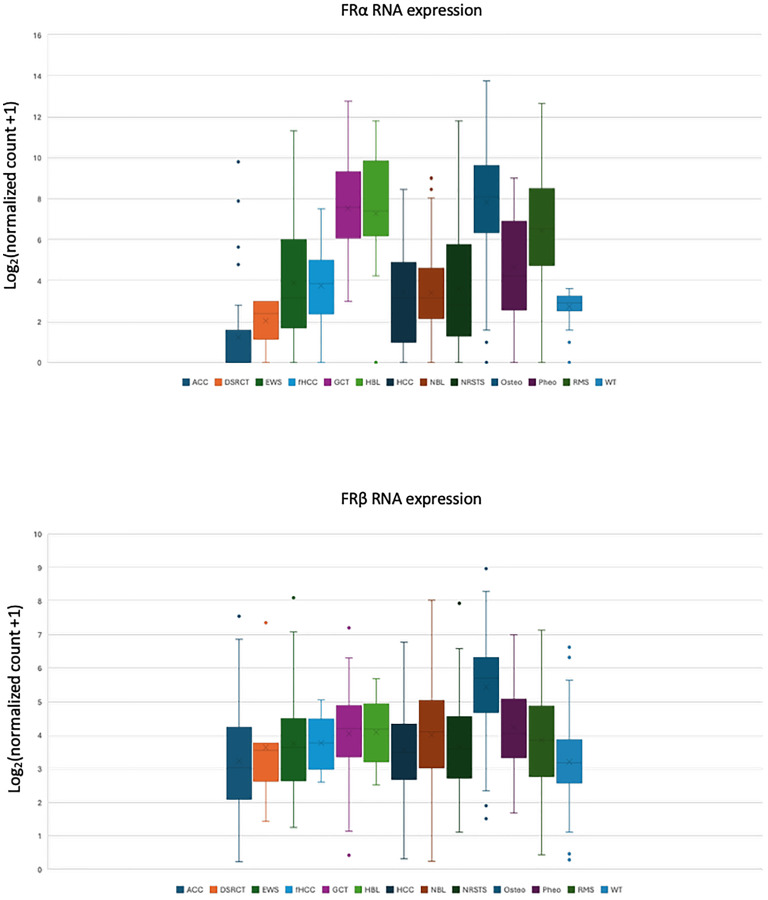
Comparison of FRα and FRβ RNA expression in pediatric tumors from the Treehouse Tumor Compendium v11 database quantified using the Kallisto log2(TPM+1) normalized approach. (A) FRα RNA expression is variable across various pediatric tumors compared to (B) FRβ RNA expression which is expressed across these same pediatric tumors. Abbreviations: ACC: Adenoid Cystic Carcinoma; DSRCT: Desmoplastic Small Round Cell Tumor; EWS: Ewing Sarcoma; fHCC: Fibrolamellar Hepatocellular Carcinoma; GCT: Germ Cell Tumor; HBL: Hepatoblastoma; HCC: Hepatocellular Carcinoma; NBL: Neuroblastoma; NRSTS: Non-Rhabdomyosarcoma; Osteo: Osteosarcoma; Pheo: Pheochromocytoma; RMS: Rhabdomyosarcoma; WT: Wilms Tumor.

In reviewing RNA expression of both FRα and FRβ, FRα demonstrated much more variability across pediatric tumors whereas FRβ had ubiquitous RNA expression across the same tumor set (Figure 4). FRα RNA expression was greatest in hepatoblastoma, germ cell tumor, osteosarcoma and rhabdomyosarcoma, which was somewhat discordant from the IHC data where the weakly expressed FRα was seen in osteosarcoma, synovial sarcoma, and Ewing sarcoma samples.

## DISCUSSION

Despite progress in medical imaging and implementation of novel adjuncts such as ICG, identification and complete resection of pediatric solid tumors and their metastases remain a challenge. Advances in fluorescence-guided surgery have shown promise for enhancing tumor identification and negative-margin excision but have been limited by the non-specific nature of ICG. Novel target-specific fluorophores such as pafolacianine have demonstrated promising use in adult ovarian and lung cancers [16]. However, the utility of pafolacianine in pediatric tumors remains unknown. In this study, we demonstrated the expression of folate receptors on a range of pediatric solid tumors, driven in large part by expression of FRβ both on the tumor and within the tumor microenvironment. This is contrast with previous literature which described FRβ as predominantly on tumor-associated macrophages [17, 18].

Pafolacianine is among the earliest of the tumor-specific agents in the burgeoning field of fluorescence-guided surgical oncology [19, 20]. Pafolacianine was originally developed to target FRα in human lung and ovarian tumors, with known cross-reactivity to FRβ. Little is known about the locations and ubiquity of FRβ expression, though recent literature supports the importance of both FRα and FRβ expression in pafolacianine use [17, 18, 21]. Our findings show that several key pediatric tumors do have modestly elevated FRα, but much more importantly, FRβ seems to be consistently expressed in pediatric tumors through a combination of tumor cell surface and within the tumor microenvironment. Importantly, the normal surrounding tissue showed little or no FRβ expression, suggesting a clinical application for delineating tumor from surrounding normal tissue. Findings on IHC of omnipresent FRβ expression in this limited set of pediatric tumor specimens is further supported by RNA-sequencing data showing elevated FRβ expression across a wide variety of pediatric tumors.

Pafolacianine was initially approved for use in ovarian cancer in 2021 after a large phase III clinical trial demonstrated a more complete resection in over half of all patients [22]. It was subsequently approved for treatment in lung cancer based on results from the phase III ELUCIDATE trial [15]. These studies were predicated on pafolacianine’s affinity for FRα and initial targets for pafolacianine trials were those with known FRα elevation [14]. Previous literature demonstrates elevated FRα expression in some pediatric tumors including: medulloblastoma, neuroblastoma, acute lymphoblastic leukemia (ALL), and osteosarcomas [23–26]. In contrast our study did not show strong FRα expression in any of the pediatric solid tumor samples. On the other hand, our study demonstrated strong FRβ expression on the majority of solid pediatric tumors. FRβ was previously only known to be expressed on tumor-associated macrophages. FRβ as the driver for reliable tumor visualization has not been previously demonstrated in pediatric tumors [17, 18]. Findings from this study exhibit ubiquitous FRβ expression, not FRα, in several pediatric tumors and within the tumor micro-environment suggesting a potential target for pan-tumor FGS with pafolacianine in pediatric surgical oncology.

Much of the recent progress in next-generation FGS has involved fluorophores conjugated to tumor-specific monoclonal antibodies that are over-expressed on specific tumor types. Examples include anti-EGFR fluorophore for pancreatic adenocarcinoma and anti-GD2 fluorophore for neuroblastoma [20]. While these tumor-specific fluorophores will certainly have an important role in FGS, “tumor agnostic” fluorophores which target cancers more universally may have even greater applicability. Findings from our study suggest that pafolacianine, via its targeting of FRβ, may have potential as a pan-tumor agent in children with extra-cranial malignancies. Another potential tumor-agnostic agent in development is Pegsitacianine, which targets the highly acidic tumor microenvironment where it releases ICG from its nanoprobe. Ongoing phase II clinical trials show promising results of Pegsitacianine in adult lung cancer and in identification of peritoneal carcinomatosis during cytoreductive surgery for multiple cancers (appendiceal, colon, pancreatic, ovarian, and mesothelioma), with potential application to other cancers [19, 27]. However, with no current data on the applicability of Pegsitacianine in pediatric patients or pediatric tumors, the folate receptor currently remains the most promising pediatric target.

Building off the encouraging results in adults with ovarian and lung tumors, and informed by this preclinical data, we have recently opened a pilot study utilizing pafolacianine in children (https://clinicaltrials.gov/ ID:NCT06235125) [28]. In this study, we are enrolling patients diagnosed with osteosarcoma, non-rhabdomyosarcoma soft tissue sarcoma (NRSTS), hepatoblastoma, rhabdomyosarcoma, Ewing sarcoma, and Wilms tumor who are undergoing either open or minimally invasive pulmonary metastasectomy. Patients will receive pafolacianine infusions prior to surgery and undergo subsequent intra-operative examination of the lungs under white light and NIR.

This pre-clinical study demonstrates a broad potential application for pafolacianine in pediatric solid tumor resection. We additionally illustrate the utility of FRβ as a ubiquitous target across various pediatric tumors. This study was limited to only deceased patients in our biorepository. Future studies will need to evaluate FR expression in pediatric tumor tissues and the tumor microenvironment as compared to normal tissue in a larger sample size and subsequently its real-world application in clinical trials.

## MATERIALS AND METHODS

### Patient sample selection

For the primary analysis, we evaluated FRα and FRβ protein expression in pediatric solid tumors by immunohistochemistry (IHC). Archived tumor samples were collected from deceased pediatric and adolescent patients diagnosed with a variety of pediatric solid tumors including: Wilms tumor, osteosarcoma, synovial sarcoma, rhabdomyosarcoma (RMS), desmoplastic small round cell tumor (DSRCT), Ewing sarcoma, and neuroblastoma (IRB#2023-6444). Samples include both primary and metastatic sites, as well as specimens obtained before and after chemotherapy treatment.

### Immunohistochemistry (IHC) and scoring

Formalin-fixed, paraffin-embedded (FFPE) tissue sections were prepared and mounted onto slides. These slides underwent deparaffinization with Discovery Wash (Ventana Medical Systems, Tuscon, AZ, USA), rehydration, and antigen retrieval as per manufacturer’s protocol with proprietary reagants as described below:

### FRα

Slides were deparaffinized on the automated system with Discovery Wash. Enzyme digestion method was used in Protease 1 solution (Ventana). FRα mouse antibody (Biocare BRI4000K AA, Concord, CA, USA) was used at a predilute concentration and incubated for 2 hours. The Ventana Omnimap Anti-mouse secondary antibody was then applied for 16 minutes. The Ventana ChromoMap DAB kit detection system was then used, and the slides were counterstained with hematoxylin. Slides were subsequently dehydrated and cover slipped per standard lab protocol.

### FRβ

Slides were deparaffinized on the automated system with Discovery Wash. Heat-induced antigen retrieval was used in CC1 solution (Ventana). The rabbit FRβ antibody (Novus Biologicals, NBP2-43654, Centennial, CO, USA) was used at a 1:800 concentration in Dako antibody diluent (Carpenteria, CA, USA) and incubated for 32 minutes. The Ventana Omnimap Anti-rabbit secondary antibody was then applied for 16 minutes. The Ventana ChromoMap DAB kit detection system was then used, and the slides were counterstained with hematoxylin. Slides were subsequently dehydrated and cover slipped per standard lab protocol.

### Quantification/scoring

Expression/Concentration intensity was quantified using a scoring system ranging from 0 to 3, where:

- 0: No immunostaining (negative expression),
- 1 (Weak): Presence of only a few focal cells with little expression,
- 2 (Moderate): More diffuse expression, but relatively weak staining, and
- 3 (Strong): Strong expression with diffuse staining.

**Figure 5:**
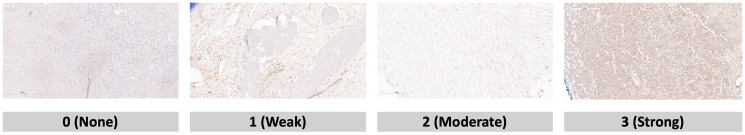
Slide scoring system created to quantify immunostaining intensity. 0 (none): No immunostaining. 1 (Weak): Presence of only a few focal cells with little expression. 2 (Moderate): More diffuse expression, but relatively weak staining. 3 (Strong): Strong expression with diffuse staining.

Scoring was performed by an experienced pathologist blinded to the clinical and treatment status of the samples to minimize bias (Figure 5). All slide examples provided in this manuscript are available at https://prism.northwestern.edu/. Full study slide set is available upon request.

### RNA sequencing database

To complement the IHC analysis, publicly available RNASeq data was obtained from the Treehouse Tumor Compendium v11 using the Xena Browser (https://xenabrowser.net/heatmap/). For each of the genes of interest (FOLR1 and FOLR2), a disease filter was applied to select expression data only from the relevant solid tumor diagnoses shown in the tables. An age filter was selected to limit data to age-at-diagnosis <30 years. Data was also limited to results with non-null expression of the gene of interest. Gene expression was reported as log2(TPM+1) normalized. The embedded Xena visualization tool was utilized to generate a box plot showing data from gene expression with subgroup samples based on the different diseases included within the filter.
